# Addressing the Challenges to Sustainable Initiatives in Value Chain Flexibility: Implications for Sustainable Development Goals

**DOI:** 10.1007/s40171-021-00288-4

**Published:** 2021-09-14

**Authors:** Ashish Dwivedi, Dindayal Agrawal, Ajay Jha, Massimo Gastaldi, Sanjoy Kumar Paul, Idiano D’Adamo

**Affiliations:** 1 Jindal Global Business School, O.P. Jindal Global University, Sonipat, India; 2 Department of Management Studies, Indian Institute of Technology, Delhi, India; 3 Jaipuria Institute of Management, Lucknow, India; 4 Department of Industrial and Information Engineering and Economics, University of L’Aquila, Via G. Gronchi 18, 67100 L’Aquila, Italy; 5 UTS Business School, University of Technology Sydney, Sydney, Australia; 6 Department of Computer, Control and Management Engineering, Sapienza University of Rome, Via Ariosto 25, 00185 Rome, Italy

**Keywords:** Cross-Impact Matrix Multiplication Applied to Classification (MICMAC), Modified total interpretive structural modeling (m-TISM), Sustainable development goals (SDGs), Value chain flexibility (VCF)

## Abstract

The value chain refers to the source of competition to facilitate organizations to maximize and sustain value for their consumers. Value chain flexibility is necessary to build sustainable initiatives in addressing ambiguity. In the literature, there is a lack of framework to highlight the challenges to sustainable initiatives in value chain flexibility. This study fills this research gap by suggesting a framework for challenges to sustainable initiatives in value chain flexibility. In this study, thirteen potential challenges to sustainable initiatives in value chain flexibility are identified and an integrated model is developed. It adopts the modified Total Interpretive Structure Model and the Cross-Impact Matrix Multiplication Applied to Classification methodology. The mixed approach is used as the modified Total Interpretive Structure Model organizes the binary interactions among the challenges, while Cross-Impact Matrix Multiplication Applied to Classification analysis organizes specific precise assessments of the driving power and dependence of the challenges. The results of the study reflect that (i) lack of supplier commitment to sustainable products and (ii) lack of knowledge toward sustainability in value chains are the challenges that achieved the highest driving power. The challenge ‘inadequate communication among the suppliers in the value chain’ is at the highest level in the analysis. The proposed framework could help government and non-government bodies to formulate policies to efficiently address challenges to sustainable initiatives in value chain flexibility.

## Introduction

The 2030 Agenda for Sustainable development, taken up by all member states of the United Nations (UN) in 2015, contributes to a common plan for peace and prosperity of society and the environment, in the present and in the future (Nellis et al., 2020). At its heart are the 17 sustainable development goals (SDGs), which are an urgent call to action by developed and developing countries in a global partnership (Mio et al., 2020). The adoption of the 2030 Agenda represents a major push to foster sustainable transition. Policies play a key role in this epochal challenge from which many opportunities for future generations are based (Costanza et al., 2016). Organizations must redefine their strategies (Singh & Sushil, 2021) and an approach that measures performance across countries is preferred (D’Adamo et al., 2021). In this context, supply chains are being reshaped to acquire green characteristics and to be competitive. Furthermore, sustainable supply chains need to build flexible capabilities to meet the challenges of the SDGs (Zhou et al., 2020).

With the advancement in globalization and the information technology (IT) revolution, supply chain management has emerged as an essential business management tool (Wongsurawat & Shrestha, 2018). In the era of globalization, where goods manufactured in one part of the country are consumed in other parts of the country, supply chains are required to be both efficient and responsive (Azaron et al., 2021). Flexibility in supply chains has contributed to the responsiveness of chains to achieve higher service levels, faster delivery, and product customization, but it leads to trade-offs in terms of higher cost and other impacts on the environment and society (Rajesh, 2020). The dimensions of flexibility include product flexibility, volume flexibility, sourcing flexibility, logistics flexibility, information flexibility, delivery flexibility, and process flexibility (Sanchez, 2005). Moreover, in the volatile, uncertain, complex, and ambiguous (VUCA) world with increased customer awareness, there is more pressure on companies to adopt sustainable practices for the betterment of the society and the environment (Worley & Jules, 2020).

Still, there are many developing or underdeveloped countries struggling to establish efficient organizational framework and infrastructure for sustainable supply chains to capture consumer taste, social and environmental impact (Sassanelli et al., 2020b). Government and citizens are called upon to be more responsible in the generation and disposal of hazardous waste, particularly toward product design and recycling (Darby & Obara, 2005; Zorpas, 2020; Zorpas et al., 2014).

The literature review reveals that several studies have focused on the requirement of flexibility to manage uncertainties, but less consideration can be seen toward the factors that focus flexibility and sustainable supply chain and their influence on SDGs. A well-defined understanding of flexibility parameters and challenges will help organizations adjust their resources in a decisive way for sustainable supply chain management. For the manufacturing sector, the SDGs are not only the potential drivers impacting the daily work routine but also the introductory ethos for the Triple Bottom Line (TBL) of sustainability (Acerbi et al., 2021; Sassanelli et al., 2020a). Another challenge to the TBL of sustainability and flexibility on supply chains is the pandemic environment created due to the coronavirus (COVID-19) outbreak. The pandemic disrupted businesses and created imbalances in poverty and availability of health facilities (Loizia et al., 2021). As global associations, regional groups, and countries plan the post-COVID19 recovery, there is a need to place the SDGs at the soul of policymaking (Elavarasan et al., 2021). Healthcare supply chains (HSCs) must be flexible and efficient to manage the challenges that have emerged in terms of matching the demand for health facilities with their supply (Yu et al., 2021a, b). In addition, the pandemic has highlighted the value of real-time information and the enormous costs of ignoring repercussions. The same is true for many sustainable indicators, where timing plays an important role in saving ecosystems and efficient administration (Bebbington & Unerman, 2018).

Sustainable development refers to meeting the needs of the present without compromising the ability of future generations (Appolloni et al., 2021; Imran et al., 2014). This study aims to focus on the challenges to sustainable initiatives in value chain flexibility (VCF) in the context of emerging economies such as India and the implications for the SDGs. Thus, in this study, the following questions are discussed:

- (a). What are the challenges to sustainable initiatives in the VCF?
- (b). What is the interrelationship among these potential challenges?
- (c). What is the approach to interpret the driving-dependence influence of each identified challenge.
- (d). What is the strength of each challenge and prioritize the challenges to sustainable initiatives in VCF?

## Literature Review

In this study, the literature survey is presented in two phases. In the first phase, the theoretical background related to sustainability and VCF is presented. The second phase highlights previous studies specific to the challenges in both VCF, and SDGs. Based on the literature survey, research gaps and contributions are acknowledged.

### Sustainability and VCF

In value chains, the flexibility component is important for improving supply chain performance while the sustainability component is important for corporate responsibility (Shen et al., 2019). In recent studies, the combination of sustainability and VCF refers to Sustainable Value Chain Flexibility (SVCF) (Bai et al., 2019). Literature assigns relevance to the relationship between sustainability and VCF. Mangano et al., (2021) suggested the sustainable value propositions for the last-mile delivery services from the perspective of retailers. The results of the study reflect the value propositions that managers and practitioners should adopt in advancing last-mile delivery Negri et al., (2021) performed a literature revoew to highlight specific studies on sustainabiity and resilience in the supply chain. Similarly, Stadnyk et al., (2021) proposed competitive business strategies for advancing the agro-industrial business network. The study’s analysis reflects that business networks should provide business process adjustments and proactive flexibility. Further, Yoo and Cho (2021) analyzed VCF’s participation in the employing green practices. The results of the study highlight that product flexibility was positively related to green practices. In addition, Taifa et al., (2020) proposed a framework for manufacturers based on the Supply Chain Operations Reference (SCOR) model considering an extended enterprise. The study identified potential Critical Success Decision Criteria (CSDC) for bulk order sharing among suppliers.

Sirilertsuwan et al., (2020) proposed a decision tool to determine tiered sourcing locations based on a number of factors. The results of the study can assist consumers in location decisions and long-term value chain planning under the triple bottom line of sustainability. Further, Yu and Solvang (2020) presented a multi-objective fuzzy-stochastic mathematical model for developing a sustainable closed-loop supply chain network design. The model helps to improve the flexibility and consistency of transportation management decision-making. Similarly, Ecer and Pamucar (2020) highlighted sustainable supply chain practices for supplier selection based on the triple bottom line of sustainability. The obtained model is tested to establish the flexibility and stability of the results considering the case of a home appliance manufacturer. Liao (2020) proposed a framework to visualize the flexibility of the value chain from the perspective of market and network coordination. The findings of the study reflect the interactions between the market and network supply chain flexibility. In addition, Han et al., (2020) proposed an analytical approach to measures the flexibility aspects of IT for logistics organizations. The analytical approach can assist organizations toward sustainable growth and flexible IT adoption.

Further, Ricciotti (2019) proposed a systematic literature review to highlight specific studies from value chain to value network. The analysis provided six potential concepts that can help organizations decide between doing business and sustainable competition. Singh et al., (2019) performed supply chain flexibility assessment by adopting the system dynamics model. Liao and Li (2019) examined internal participation and external capability to improve the organization's innovation capability. Alonso-Muñoz et al., (2020) examined involvement in group companies as a source of external knowledge to achieve large profitable innovations. The present study contributes to the literature by investigating internal involvment and external capability in developing sustainable innovation capabilities. In addition, Sahu and Kohli (2019) proposed a model that includes sustainable practices for flexibility in drug delivery. This proposed model is value-added with an identification technique of weak and strong sustainable practices. A case study for a pharmaceutical organization is presented to reflect the real-life application of the study.

Bag et al., (2018) examined supplier relationship management, flexibility, and innovation on sustainable supply networks. The study adopted Integrating Institutional Theory (IT) and Resource-Based View (RBV) theory to perform the examinations. Further, Yu and Solvang (2018) sug gested a two-stage mixed-integer programming model for network design of sustainable reverse logistics systems under uncertainty. The objectives of the study are Pareto solutions between profitability and environmental performance. Ansari and Kant (2017) performed a literature review to highlight previous studies specific to sustainable supply chains.

### Challenges in both VCF and SDGs

Another piece for our study is to identify what the literature reports in terms of changes for both VCF and SDGs. Häger et al., (2021) proposed a case study to help coffee producers overcome the threat of economic and environmental challenges inferred from the manufacturing layout of agrochemical operations. The study considered the socioeconomic, environmental, and psychological perspectives of sustainability. Similarly, Weersink et al., (2021) highlighted the effects of the COVID-19 pandemic on the agri-business system. The pandemic led to product diversification by providing more alternatives for flexibility management and has less impact on the SDGs. In addition, Tan et al., (2021) adopted the business model canvas to study market positions under the changing industry landscape for coffee processors. The results of the study reflect that different business models have different priorities in response to value chains. Further, Chirra et al., (2020) examined the barriers to flexibility from a sustainable perspective in the context of automotive supply chains. The study contributes to the current literature by linking the supply chain flexibility, sustainability, and sales promotions. Kumar and Anbanandam (2020) per formed a situation–actor–process–learning–action–performance analysis to improve understanding toward supply chain resilience.

Contador et al., (2020) identified challenges in building a flexible industry in the context of Brazilian manufacturing organizations. The study highlights potential opportunities based on Industry 4.0 to build a flexible industry. Further, Pérez-Pérez et al., (2019) suggested a conceptual model for manufacturing and supply chain flexibility. The study suggests a framework that assists the managers in improving VCF. Also, Majumdar and Sinha (2018) analyzed the potential barriers of green supply chain management in the context of Small and Medium Enterprises (SMEs). The findings of the study will help prepare SMEs for sustainability by removing potential barriers.

Similarly, Bechtsis et al., (2018) provided a framework that supports the effective assimilation of Intelligent Autonomous Vehicles (IAVs) into sustainable supply networks. Agarwal et al., (2018) presented literature specific to global manufacturing value chains, smart specialization, and flexibility. Further, Nauhria et al., (2018) developed a framework for a strategic value chain in the context of the automotive manufacturing industry. The results of the study can be used to formulate a comprehensive strategy from the perspective of the automotive manufacturing industry. Harnesk et al., (2017) proposed a study to regulate a global value chain through the adoption of sustainability criteria. The study was conducted in the context of a liquid transport biofuel region originating in Sweden. Similarly, Boström et al., (2015) explored specific governance challenges that hinder global supply chains from becoming sustainable. The results of the study provide convenience to encourage sustainable governance. Mangla et al., (2014) suggested a framework to study the interactions among factors that influence sustainable supply chains at risk.

van Keulen and Kirchherr (2021) identified barriers and enablers in the coffee value chain. The study focused on the implementation of circular economy concepts. Werning and Spinler (2020) identified barriers in the transition to circular economy along the value chain. Prioritization of barriers is performed in the study. In addition, Chen et al., (2020) performed a study to discuss the relationship between value chain and environmental cost control. In this study, artificial intelligence decision tree algorithm was applied. Similarly, Sarc et al., (2019) performed a literature review to highlight studies specific to digization and smart robotics in the circular economy value chain.

### Identification of Challenges to Sustainable Initiatives in VCF

Based on the literature review and interaction with specific experts from industry and academia, the following thirteen potential challenges to sustainable initiatives in the VCF are recognized as reflected in Table 1.

**Table 1:** Challenges to sustainable initiatives in VCF

Code	Challenges to sustainable initiatives in VCF	References
B1	Lack of consumer orientation toward sustainability in value chain	Shibin et al. (2016)
B2	Lack of distribution flexibility in the value chain	Singh et al., (2017); Tan (2021)
B3	Lack of supplier commitment towards sustainable products	Lu et al., (2018); Horva´th and Szabo´ (2019); Chirra et al., (2020); Pratap et al., (2021)
B4	Lack of knowledge towards sustainability in value chain	Bolwig et al., (2010); Chirra et al., (2020)
B5	Lack of IT integration in value chain	Gunasekaran and Ngai (2004); Rosca and Bendul (2019)
B6	Insufficient government rules towards sustainable initiatives	Birkel et al. (2019); Contador et al., (2020); Chowdhury et al., (2021); Paul et al., (2021)
B7	Financial constraints towards sustainable initiatives	Kushwaha and Sharma (2016); Luthra and Mangla (2018); Patil et al., (2021)
B8	Capacity constraints in value chain flexibility	Gosling et al. (2010); Kazemian and Aref (2016)
B9	Lack of trust in the value chain	Singh et al., (2017); Macready et al., (2020)
B10	Inadequate Information sharing in value chain	Du et al., (2012); Kazemian and Aref (2016); Paul and Chowdhury (2020)
B11	Lack of top management commitment towards flexibility in value chain	Raut et al., (2018); Raj et al. (2019)
B12	Lack of manufacturing flexibility in the value chain	Mishra et al. (2014); Wei et al., (2017); Paul and Chowdhury (2021)
B13	Inadequate communication among the suppliers in the value chain	Fearne et al., (2012); Shibin et al., (2016)

## Research Methodology

### Data Collection and Questionnaire Design

In this study, a structured questionnaire was prepared for the potential challenges for sustainable initiatives in VCF. The questionnaire was personally brought to the respondent to avoid unattended emails and to ensure that the targeted expert was the respondent. The questionnaire was formulated in English. The targeted manufacturing industries were located in India’s National Capital Region (NCR).

### m-TISM Methodology

Interpretive Structure Modeling (ISM) is a computer-aided methodology coined in 1973 by Warfield. ISM methodology is used to generate interactions among different elements originating from a specific condition. In ISM, expert recommendations make a decision concerning the interactions among elements. Therefore, its nature is interpretative (Yadav et al., 2020). In addition, the ISM approach can investigate a variety of management situations through its ordered model description, but this approach has some disapprovals (Dwivedi & Madaan, 2020;  Dwivedi et al., (2020); Mathivathanan et al., 2021). To conquer the short aspects of the ISM approach, an extended ‘TISM’ model was presented. In the TISM technique, the limitations of ISM are emphasized by using the ‘Interpretive Matrix’ where causal reasoning is limited to the time of data collection by experts and is illustrated in the matrix (Sushil, 2017). Therefore, in TISM, explanations of relationships are highlighted next to the corresponding links connecting the pair of items. Fewer experts are needed in m-TISM methodology compared to some other multicriteria decision-making (MCDM) techniques such as analytical hierarchical process, decision-making trial, and evaluation laboratory, and Delphi (Chowdhury & Paul, 2020). A number of studies have employed the TISM approach to solve complex problems. Chaudhury et al., (2021) suggested a framework for critical success factors of digital supply chains by adopting a TISM approach. Lakshmi et al., (2020) identified factors influencing the epidemiological characteristics of the pandemic. Dhir and Dhir (2020) modeled the enablers of strategic thinking by adopting a TISM approach. Dwivedi et al., (2019) suggested Key Performance Indicators (KPIs) for sustainable manufacturing considering the case of leather industry. The m-TISM (Dhir et al., 2021; Rajan et al., 2021; Sushil, 2018) is the extension of TISM (Sushil, 2012) to the knowledge of interactions, the degree of association, and the logic behind the interactions. The basic steps taken in the m-TISM approach are shown below:

*Step I: Identifying challenges to sustainable initiatives in VCF*

Identifying challenges that are compelling to the situation is the first step of m-TISM methodology (Kumar et al., 2018). A total of thirteen challenges are extracted from the study specific to sustainable initiatives and VCF from literature review, surveys, and interviews as highlighted in (Table 1).

*Step II: Defining contextual interactions*

Contextual interactions among the potential challenges obtained must be defined to generate a framework (Kumar et al., 2019). In this study, contextual interactions among challenges to sustainable initiatives in VCF are inferred. A questionnaire survey was conducted among several professionals specific to their expertise in sustainability and value chains.

*Step III: Interpretation of interactions*

Interpretation of the interactions among the identified potential challenges is conducted to understand the consistency of the model (see Appendix A).

*Step IV: Pair-wise comparison*

An interaction among the challenges with a presence or non-presence of interaction matrix is obtained from the experts’ suggestions in Table 2.

**Table 2:** Initial Reachability Matrix for challenges to sustainable initiatives in VCF

Code	B1	B2	B3	B4	B5	B6	B7	B8	B9	B10	B11	B12	B13
B1	1	0	0	0	0	1	1	1	1	1	1	1	1
B2	0	1	0	0	0	1	1	1	1	1	1	1	1
B3	0	0	1	1	1	1	1	1	1	1	1	1	1
B4	1	1	1	1	1	1	1	1	1	1	1	1	1
B5	0	0	0	0	1	1	0	1	1	1	1	1	1
B6	0	0	0	0	0	1	1	0	1	1	0	1	1
B7	0	0	0	0	0	0	1	0	1	1	0	1	1
B8	0	0	0	0	0	1	0	1	1	1	1	1	1
B9	0	0	0	0	0	1	1	0	1	1	0	1	1
B10	0	0	0	0	0	0	0	0	1	1	0	1	1
B11	0	0	0	0	0	0	0	0	0	0	1	1	1
B12	0	0	0	0	0	0	0	0	0	0	0	1	1
B13	0	0	0	0	0	0	0	0	0	0	0	0	1

*Step V: Obtaining the Reachability Matrix and transitivity check*

**Fig. 1:**
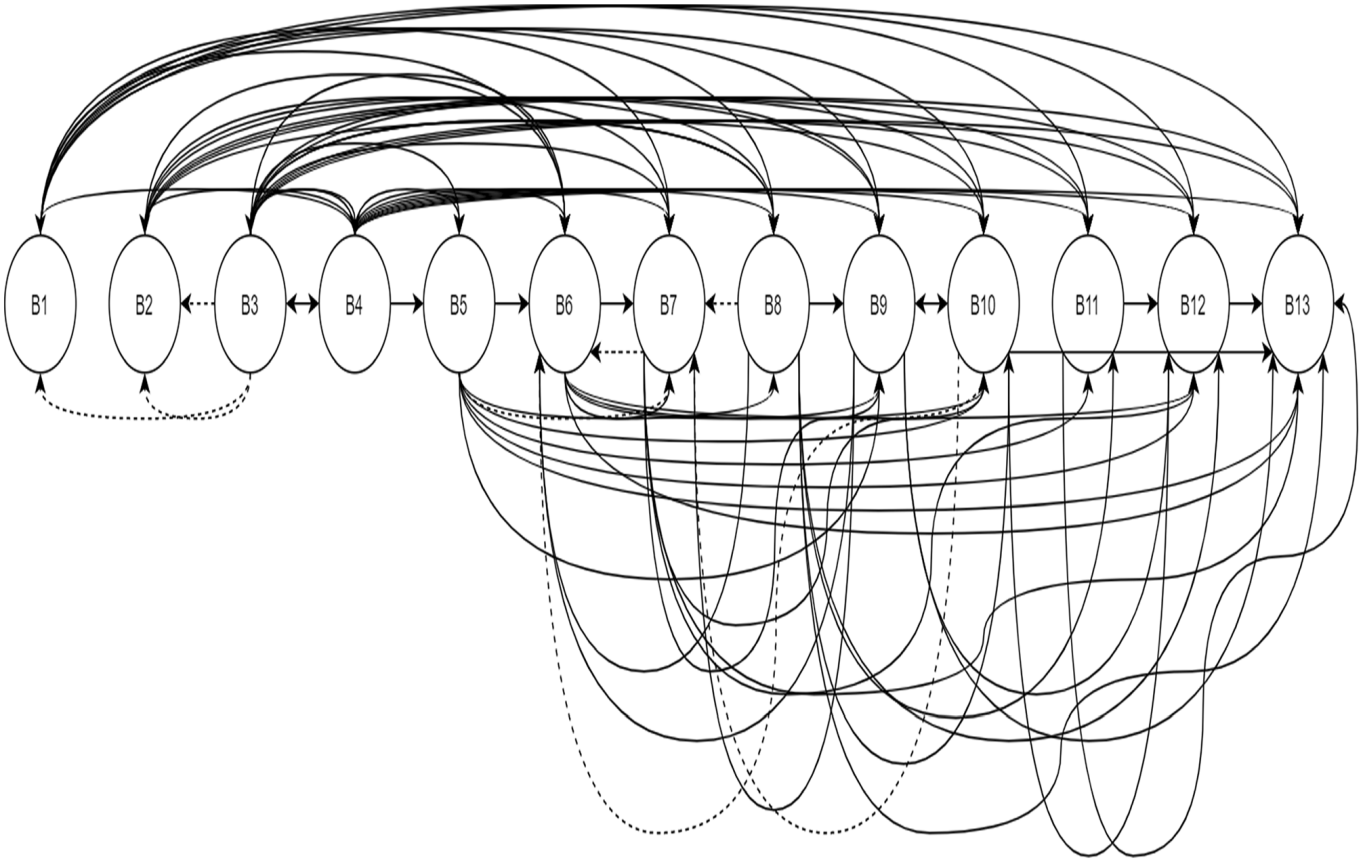
Diagraph reflecting interactions between the challenges to sustainable initiatives in VCF

The initial reachability matrix for the identified potential challenges is arranged in Table 2. In addition, the reachability matrix is checked for updated transitivity links updated as 1* to produce the final reachability matrix in Table 3. Simultaneously, transitivity is checked. Figure 1 highlights the simultaneous control of transitivity. In m-TISM, the basic steps of TISM I, II, III, IV and V are combined, where along with pair-wise comparison, simultaneous transitivity checking is performed.

**Table 3:** Final Reachability Matrix for challenges to sustainable initiatives in VCF

Code	B1	B2	B3	B4	B5	B6	B7	B8	B9	B10	B11	B12	B13	Dr
B1	1	0	0	0	0	1	1	1	1	1	1	1	1	**9**
B2	0	1	0	0	0	1	1	1	1	1	1	1	1	**9**
B3	1*	1*	1	1	1	1	1	1	1	1	1	1	1	**13**
B4	1	1	1	1	1	1	1	1	1	1	1	1	1	**13**
B5	0	0	0	0	1	1	1*	1	1	1	1	1	1	**9**
B6	0	0	0	0	0	1	1	0	1	1	0	1	1	**6**
B7	0	0	0	0	0	1*	1	0	1	1	0	1	1	**6**
B8	0	0	0	0	0	1	1*	1	1	1	1	1	1	**8**
B9	0	0	0	0	0	1	1	0	1	1	0	1	1	**6**
B10	0	0	0	0	0	1*	1*	0	1	1	0	1	1	**6**
B11	0	0	0	0	0	0	0	0	0	0	1	1	1	**3**
B12	0	0	0	0	0	0	0	0	0	0	0	1	1	**2**
B13	0	0	0	0	0	0	0	0	0	0	0	0	1	**1**
De	**3**	**3**	**2**	**2**	**3**	**10**	**10**	**6**	**10**	**10**	**7**	**12**	**13**	

1 means direct relations; 1* means Transitive relations

De- Dependence, Dr- driving power

**Table 4:** Iteration 1 for level partitioning

Code	Reachability Set	Antecedent Set	Intersection Set	Level
B1	1,6,7,8,9,10,11,12,13	1,3,4	1	
B2	2,6,7,8,9,10,11,12,13	2,3,4	2	
B3	1,2,3,4,5,6,7,8,9,10,11,12,13	3,4	3,4	
B4	1,2,3,4,5,6,7,8,9,10,11,12,13	3,4	3,4	
B5	5,6,7,8,9,10,11,12,13	3,4,5	5	
B6	6,7,9,10,12,13	1,2,3,4,5,6,7,8,9,10	6,7,9,10	
B7	6,7,9,10,12,13	1,2,3,4,5,6,7,8,9,10	6,7,9,10	
B8	6,7,8,9,10,11,12,13	1,2,3,4,5,8	8	
B9	6,7,9,10,12,13	1,2,3,4,5,6,7,8,9,10	6,7,9,10	
B10	6,7,9,10,12,13	1,2,3,4,5,6,7,8,9,10	6,7,9,10	
B11	11,12,13	1,2,3,4,5,8,11	11	
B12	12,13	1,2,3,4,5,6,7,8,9,10,11,12	12	
B13	13	1,2,3,4,5,6,7,8,9,10,11,12,13	13	1

*Step VI: Segregating of the reachability matrix*

The final reachability matrix obtained is segregated into various levels by conducting a number of iterations for each identified potential challenge and the levels are reached in Tables 4 and 5.

**Table 5:** Iterations (1–6 consolidated) for level partitioning

Code	Reachability Set	Antecedent Set	Intersection Set	Level
B1	1	1,3,4	1	5
B2	2	2,3,4	2	5
B3	3,4	3,4	3,4	6
B4	3,4	3,4	3,4	6
B5	5	3,4,5	5	5
B6	6,7,9,10	1,2,3,4,5,6,7,8,9,10	6,7,9,10	3
B7	6,7,9,10	1,2,3,4,5,6,7,8,9,10	6,7,9,10	3
B8	8	1,2,3,4,5,8	8	4
B9	6,7,9,10	1,2,3,4,5,6,7,8,9,10	6,7,9,10	3
B10	6,7,9,10	1,2,3,4,5,6,7,8,9,10	6,7,9,10	3
B11	11	1,2,3,4,5,8,11	11	3
B12	12	1,2,3,4,5,6,7,8,9,10,11,12	12	2
B13	13	1,2,3,4,5,6,7,8,9,10,11,12,13	13	1

*Step VII: Development of the digraph*

A simple digraph showing the transitive interactions is obtained and presented in Fig. 1.

*Step VIII: Obtaining the m-TISM*

**Fig. 2:**
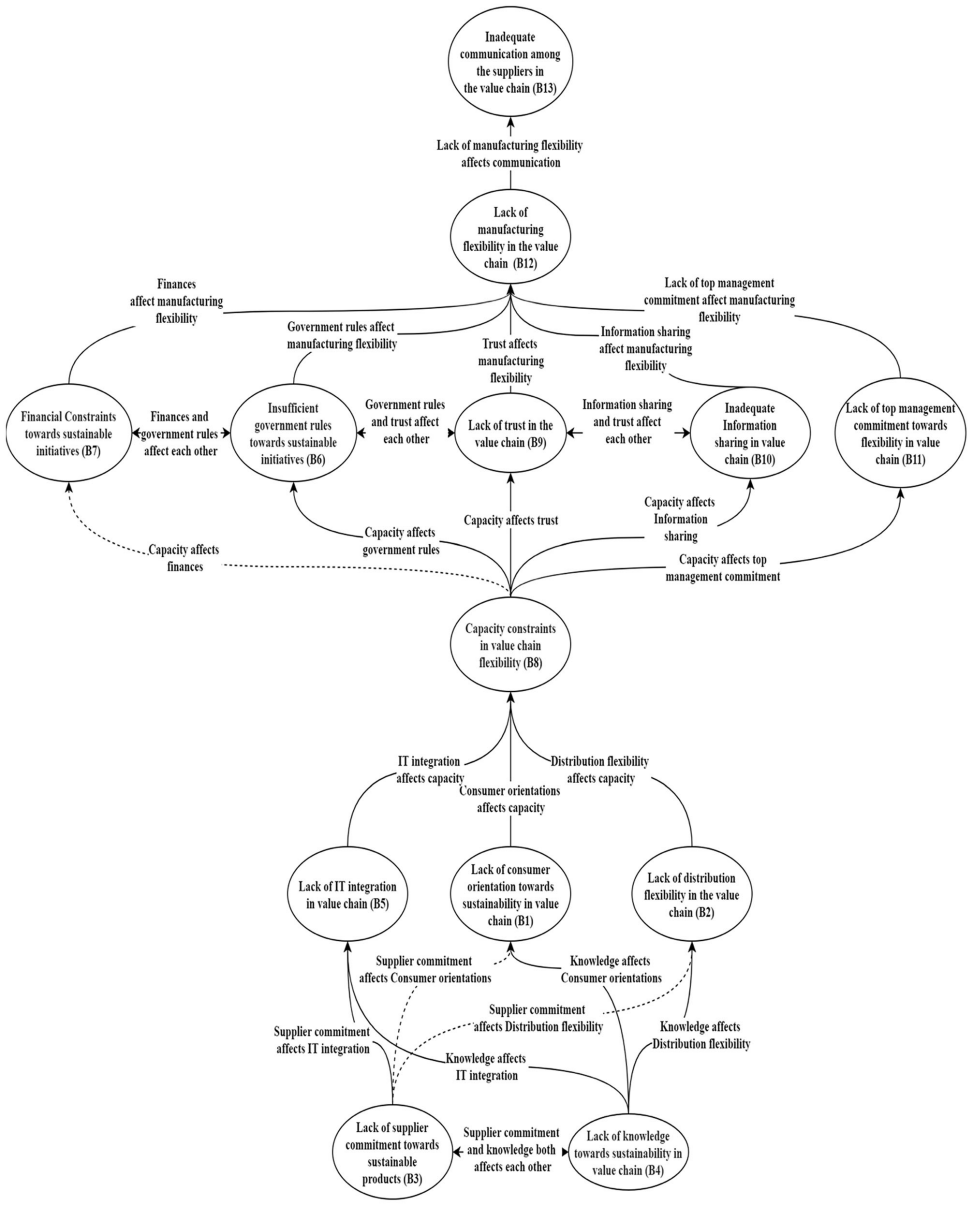
m-TISM Model presenting interrelationships among the challenges to sustainable initiatives in VCF

A m-TISM model is obtained from the digraph that defines the interrelationships among the identified potential challenges as reflected in Fig. 2. The bold arrow represents the direct link while the transitive link is represented by the dashed arrow.

### Development of m-TISM Model for the Identified Challenges

#### Developing the Initial Reachability Matrix

In m-TISM, an initial reachability matrix is obtained by substituing 1 and 0 according to a set of instructions. The initial reachability matrix is highlighted below in Table 2.

#### Developing the Final Reachability Matrix

The final reachability matrix is obtained by integrating the transitivity presented by ‘‘*’’ according to the transitivity rule as shown in Table 3.

#### Level Partitions

The achievability and set of antecedents for each identified potential challenge are obtained from the final achievability matrix. The procedure is repeated until all challenges reach their respective levels. The iterations are depicted in Tables 4 and 5.

### MICMAC Analysis

**Fig. 3:**
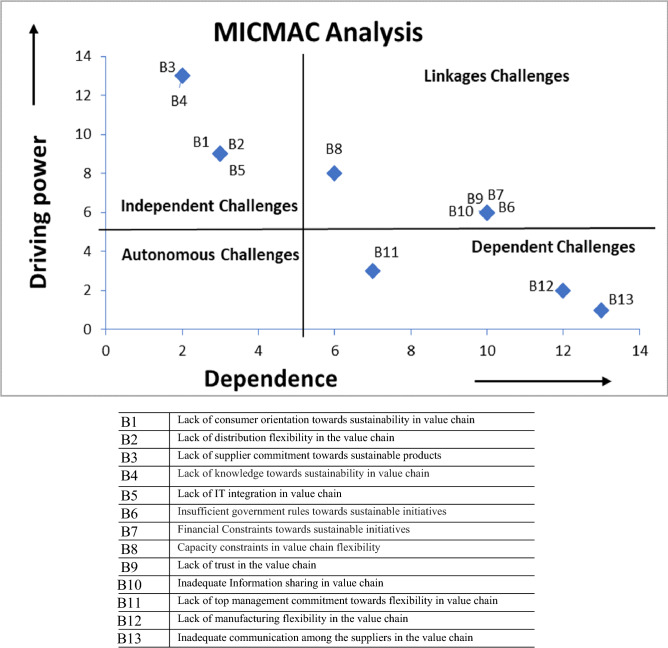
Challenges to sustainable initiatives in VCF reflecting driving power and dependence

MICMAC analysis is obtained from the final reachability matrix and is used to define the driving power and dependence on the interrelated challenges (Mishra et al., 2017). Based on the analysis, the challenges are distributed into four different categories:

- *Autonomous challenges:* The identified challenges that include weak driving power and dependence are categorized under the first quadrant. In this study, there are no autonomous challenges from our identified potential challenges (see Fig. 3).
- *Dependent challenges:* Identified challenges that have weak guidance but strong dependence are categorized under the second quadrant. From the obtained list, challenges such as ‘lack of top management commitment toward flexibility in value chain (B11)’, ‘lack of manufacturing flexibility in the value chain (B12) and ‘Inadequate communication among the suppliers in the value chain (B13)’ are posed as dependent challenges because they represent strong dependence but relatively weak driving power.
- *Linkage challenges:* The identified challenges that have high dependence and high driving power are categorized under the third quadrant. In this study, linkage challenges among our identified potential challenges are ‘insufficient government rules toward sustainable initiatives (B6)’, ‘financial constraints toward sustainable initiatives (B7)’, ‘capacity constraints in value chain flexibility (B8)’, ‘lack of trust in the value chain (B9)’ and ‘inadequate information sharing in value chain (B10)’(see Fig. 3).
- *Independent challenges:* Identified challenges that have strong driving power but weak dependence are categorized under the fourth quadrant. In this study, challenges such as ‘lack of consumer orientation toward sustainability in value chain (B1)’, ‘lack of distribution flexibility in the value chain (B2)’, ‘lack of supplier commitment toward sustainable products (B3)’, ‘lack of knowledge toward sustainability in value chain (B4)’, and ‘lack of IT integration in value chain (B5)’ are classified as independent challenges because they reflect strong driving power but weak dependence. The construct for the dependence and driving power analysis is presented in Fig. 3.

## Results, Discussions, and Implications

This study is based initially on a literature review aimed at identifying sustainable initiatives and then a series of meetings with professionals to define any interrelationships among these initiatives (m-TISM approach) evaluating their driving power and dependence analysis. The obtained m-TISM model can be studied in six different levels. The challenge ‘inadequate communication among the suppliers in the value chain (B13)’ is at the first level. The placement of this challenge at the first level reflects that it is influenced by the other challenges identified in this study. The challenge ‘lack of manufacturing flexibility in the value chain (B12)’ is placed at the second level. ‘Insufficient government rules toward sustainable initiatives (B6)’, ‘financial Constraints toward sustainable initiatives (B7)’, ‘lack of trust in the value chain (B9)’, ‘inadequate information sharing in value chain (B10)’ and ‘lack of top management commitment toward flexibility in value chain (B11)’ are the challenges found at the third level. The challenge ‘capacity constraints in value chain flexibility (B8)’ is located at the fourth level. Challenges such as ‘lack of consumer orientation toward sustainability in value chain (B1)’, ‘lack of distribution flexibility in the value chain (B2)’ and ‘lack of IT integration in value chain (B5)’ are placed at the fifth level. The challenges such as ‘lack of supplier commitment toward sustainable products (B3)’ and ‘lack of knowledge toward sustainability in value chain (B4)’ are placed at the sixth and final level in the m-TISM diagram (Fig. 2). In the m-TISM diagram, the direction of the influence of one challenge on another challenge is represented by the direction of the arrowhead. In addition, the driving power and dependence for identified potential challenges can be obtained using the MICMAC analysis. Challenges to sustainable initiatives in VCF are segregated into four different groups (autonomous, linkage, dependent and independent) by adopting the MICMAC tool (Fig. 3). The results of the MICMAC analysis facilitate managers and practitioners to visualize and judge the influence of each identified challenge.

The sustainability challenge is part of an international framework, and without the support of the group of 7 (G7), an informal group of seven nations (USA, Canada, France, Germany, Italy, Japan, and the UK), it would not be possible to achieve the goal. In fact, the distribution of pollutant emissions is not the same in all parts of the world. Therefore, greater responsibility is needed on the part of industrialized countries, but also that emerging countries do not benefit from distorted market advantages. This study aims to provide support for emerging countries to identify how sustainability can be seen as a competitive advantage for their development. Furthermore, it emerges from the literature how initiatives that are able to combine sustainability and resilience will be able to overcome the problems generated by the pandemic period (Alonso-Muñoz et al., 2021; D’Adamo et al., 2020a; Mohammed et al., 2021).

Analyzing a business perspective, some authors show that risk management culture, supply chain flexibility, and internal integration are able to increase the financial performance of firms through resilience efforts (Chunsheng et al., 2020). In addition, internal and external sources of change require the adoption of a dynamic model in which flexibility can play a key role. Flexibility is the ability of a company to respond to changes in the environment, technology, organization, and strategy both quickly and at a low cost. Thus, it consists of initiatives geared toward improving also efficiency and organizational performance (Shukla et al., 2019). In particular, the optimization process is oriented toward assessing the best economic solution (Sushil, 2015). The relationship between SDGs and flexibility requires that this optimization also takes into account the social and environmental side. The interaction between sustainable models and Industry 4.0, which aims to foster the automation and digitization of production systems, leads companies to rethink their strategies by identifying new business models (Rocca et al., 2020). The current amount of funding and investment related to the SDGs is considered to be less than what is needed, and this appears to be particularly true in developing countries (Barua, 2020).

The literature pays attention to the definition of appropriate evaluation methodologies aimed at assessing different flexible initiatives (Sushil, 2018). A conceptual framework emphasizes that sustainable supply chain flexibility increases in correspondence with managers' environmental attitudes and when managers' cognitive style is intuitive (Yu et al., 2021a, b). This study confirms these previous analyses and identifies a framework capable of subdividing the different initiatives according to both driving power and dependence.

The key findings of this study emphasize that two challenges out of all are the ones that can achieve the highest driving power: lack of supplier commitment toward sustainable products and lack of knowledge toward sustainability in value chain. In fact, sustainable initiatives require a change not only in the way of doing business but also in the way of managing the public good. Climate change is objective evidence, and initiatives aimed at developing new economic models and strategies based on the green economy, the circular economy, and the bioeconomy represent a challenge that cannot be ignored by anyone, especially by governments. Consequently, in the presence of insufficient government rules toward sustainable initiatives, there is a strong risk of penalizing not only the present development of an area but also its future.

Sustainable optimization is based on the principle of proximity with supply chains that should be shortened to reduce the environmental impact of infrastructures. However, the balance between supply and demand with economic profit may be preferred. It is difficult to change this principle, which is the basis of doing business. However, it is necessary to communicate the advantages associated with the use of natural resources, of working in conditions of minimum risk to the health of citizens, of no exploitation of people. In fact, where the organization and the worker have the same objective, the benefits translate directly into the well-being of the company and the ability to generate income for the entire community. Inadequate communication among the suppliers in the value chain deriving by information asymmetries would lead to a loss of value for all the shareholders.

The transition from a fossil-based economy to one in which renewable and circular resources play a decisive role is clearly linked to investments and related subsidies policy (Loizia et al., 2019; Zorpas et al., 2021). The regional economies that have benefited from these tools are characterized by the technological development of environmentally friendly plants, a growth in the number of plants installed, and the training of professional skills (Appolloni et al., 2021; D’Adamo et al., 2020b). There is also a greater awareness among people that recognize a green premium and/or a circular premium. However, there is a gap between attitude and behavior, as people struggle to transform their sustainability ideas and propensities into purchasing decisions. Often financial constraints toward sustainable initiatives are a barrier that does not allow to reach such development goals. A policy of subsidies, which are granted according to the actual level of sustainability associated with actions and practices in which the environmental benefit is quantified, must be flanked by a policy of taxation on what pollutes and on what causes serious damage to human health. So green finance would appear to be decisive support, as would a significant increase in the cost of CO_2_ compared to current values (in recent years we have gone from 20 €/tonne to 40 €/tonne).

This study confirmed how flexibility moves toward the SDGs. Previous studies highlight the positive relationship between sustainability and resilience within decision-making processes in risk measurement (Settembre-Blundo et al., 2021); of the potential benefits associated with the implementation of a flexible consumption tax (Gerbeti, 2021); of the decisive contribution of renewable energy globally (Ikram, 2021); and in the resilience of the education sector following pandermia (Ahmed et al., 2021).

The theory of human relations has highlighted the need for a proper balance between the individual and the organization, in which the individual evolves and seeks to achieve his or her own balance and individual learning comes to fruition when it is transformed into organizational learning. Top management tends to indicate the direction to be followed in all leadership styles (participative, consultative, paternalistic, and authoritarian) and therefore its lack of commitment to flexibility in the value chain would be a disincentive to the whole organization. It is therefore essential to be able to identify opportunities even when the surrounding events are negative.

Finally, the pandemic period has highlighted the fragility of human beings, but it can also be seen as a wake-up call that all 17 SDGs goals are relevant and that the concept of sustainability is based on overcoming selfishness and adaptability. In this framework, it is necessary to be able to react to shocks in order to resume the original form (resilience) and to be able to change for the better (flexibility) in order to seize opportunities. This is only possible if there is a real collaboration among all countries for substantial support to poor regions.

## Conclusions, Limitations and Future Research Directions

Climate change is a global urgency and the scientific literature has the ambitious goal of indicating strategies, actions, and responses. This work confirms first of all that VCF is necessary to build sustainable initiatives. Markets are characterized by an increasingly turbulent environment caused by growing global competition, technological change, and changing customer expectations. In addition to these aspects, there is also the socioeconomic crisis brought about by COVID-19. Flexibility is that mechanism that is necessary for every enterprise and is an order-winning factor in various markets. In geographical areas where a regulatory system has been applied that favors only sustainable solutions, companies that have not modernized will risk exiting their market. This will be even more intense if the regulatory framework plans to penalize fossil-based products and activities. Our results identify clearly as lack of knowledge toward sustainability in value chain is the challenge characterized by the highest driving power.

Businesses that incorporate sustainability principles into their strategies and practices can be competitive in the local market by fostering the development of a local supply chain. This process includes the creation of smart networks in which companies share their resources in order to be competitive in a global market. The results show that inadequate communication among the suppliers in the value chain has among the lowest driving power. In addition, governments using public money to encourage the use of resources with a high environmental impact would risk favoring investments with only short-term effects, since in all sectors demand is very green and therefore supply should be able to meet it.

This study is limited to thirteen challenges to sustainable initiatives in the VCF. More challenges can be identified for future research, and software such as analysis of a moment structures (AMOSs) can be adopted to facilitate the analysis and explore the interactions among the identified challenges. In particular, the impact of the growing attention of younger generations to these issues should be explored. In this way, the combination of innovation and sustainability can result in the development of a Blue Ocean Strategy. SDG12 will be highly influenced by this strategic approach.

There are three directions for future research. The first is to move from a macro- to a micro-analysis in which the relationship between VCF and sustainability is assessed at the level of each individual company for a given sector. Also assessing any points of contact between sectors to create synergies particularly where industrial clusters can be created. The second is to replicate this study in other territorial contexts and assess possible convergences (e.g. industrial symbiosis). Similarly, the contribution that should come from more developed countries should also be included in the analysis. In this perspective, a key role will be to consider policies to define which ones are capable of promoting sustainable transition. The third concerns a different panel of experts that could lead to different indications. The goals of the SDGs are the blueprint toward which governmental and non-governmental forms aim to go, and real change will occur in those contexts where resilience and flexibility are in harmony by opting for sustainable initiatives.

Key Questions

1. Is value chain flexibility really important for building sustainable initiatives?
2. What is the role of flexibility towards sustainable production and consumption?
3. What is the role of flexibility within sustainable organizational models?

## Appendix A: Interpretive matrix for the challenges to sustainable initiatives for VCF

**Table:**

S.no.	1	2	3	4	5	6	7
1							
2							
3	Supplier commitment affects Consumer orientations	Supplier commitment affects Distribution flexibility		Supplier commitment affects knowledge	Supplier commitment affects IT integration		
4	Knowledge affects Consumer orientations	Knowledge affects Distribution flexibility	Knowledge affects supplier commitment		Knowledge affects IT integration		
5							
6							Government rules affect Finances
7						Finances affect government rules	
8						Capacity affects government rules	Capacity affects finances
9						Trust affects government rules	
10							
11							
12							
13							

S.no.	8	9	10	11	12	13
1	Consumer orientations affects capacity					
2	Distribution flexibility affects capacity					
3						
4						
5	IT integration affects capacity					
6		Government rules affect trust			Government rules affect manufacturing flexibility	
7					Finances affect manufacturing flexibility	
8		Capacity affects trust	Capacity affects Information sharing	Capacity affects top management commitment		
9			Trust affects Information sharing		Trust affects manufacturing flexibility	
10		Information sharing affects trust			Information sharing affect manufacturing flexibility	
11					Lack of top management commitment affect manufacturing flexibility	
12						Lack of manufacturing flexibility affects communication
13						
